# Patient demographics, diagnoses, and care needs in a Norwegian Community Virtual Ward versus Skilled Nursing Facility: a longitudinal comparative cohort study

**DOI:** 10.1186/s12913-025-13827-x

**Published:** 2025-12-02

**Authors:** Emma Sigridsdatter Jones, Sine Maria Herholdt-Lomholdt, Annelise Norlyk, Francis Odeh

**Affiliations:** 1 Bodø Municipality, Kongens gate 23, Bodø, 8006 Norway; 2 Faculty of Nursing and Health Sciences, Nord University, Universitetsallèen 11, Bodø, 8026 Norway; 3 Department of Public Health, Nursing and Healthcare, Aarhus University, Bartholins Alle 2, Aarhus C, 8000 Denmark; 4 Department of Community Medicine, UiT: The Arctic University of Norway, Postboks 6050, Stakkevollan, Tromsø, 9037 Norway

**Keywords:** Primary healthcare, Municipal healthcare services, Virtual ward, Community virtual ward, Skilled nursing facility, Frailty, Patient characteristics

## Abstract

**Background:**

Since the early 2000s, Virtual Wards have referred to models where patients receive care at home rather than in traditional healthcare facilities. In 2021, the municipality of Bodø, Norway established a Community Virtual Ward (CVW) model, offering enhanced medical follow-up for older home residents. Unlike previous models, this CVW is fully municipality operated, features broad inclusion criteria, and includes a dedicated physician. The initiative responds to challenges related to longevity, frailty and task-shifting from hospitals to primary care. This study aimed to compare patient demographics and care needs between a CVW and a traditional Skilled Nursing Facility (SNF), and to access whether the CVW model can serve as a viable alternative to institutional care.

**Methods:**

A longitudinal comparative cohort study was conducted with 55 participants transitioning from hospital to either a CVW or a SNF. Data on demographics, frailty, functional independence, and service utilization were collected at admission, discharge, and three months post-discharge. Descriptive and regression analyses were used to identify predictors of recovery and service needs.

**Results:**

CVW patients were younger, had fewer chronic conditions, lower frailty levels, and shorter admission periods compared to SNF patients. Across both groups, baseline frailty and functional independence at discharge were key determinants of recovery and service utilization 90 days post-discharge. Differences between groups were statistically significant for frailty, functional scores, and length of stay. Comorbidities were associated with smaller improvements during the inpatient stay but did not predict longer-term recovery.

**Conclusion:**

The findings suggest that the CVW model is a viable alternative to short-term institutional care for mildly frail patients who benefit from rehabilitation-focused, home-based services. SNFs remain essential for frailer individuals requiring intensive support. The results highlight the need for systematic frailty assessment in primary care to optimize care pathways for aging populations.

## Background

Longevity stands among society’s greatest achievements but also presents major medical and socio-demographic challenges in the 21st century. By 2050, nearly two billion people will be aged 60 or older, with 425 million aged 80 or above [1]. Frailty, a multidimensional syndrome linked to age-related physiological decline, is a leading cause of morbidity and mortality among older adults [2, 3]. Despite its recognized importance, there is no universally agreed-upon definition. A key feature of frailty is increased vulnerability to health deterioration triggered by minor stressors [3, 4]. Frailty is associated with adverse health outcomes, including falls, hospitalization, delirium, disability, and institutionalization [5–7] and requires holistic, multidisciplinary approaches that address the complexity of patients’ needs [3] and the maintenance of functional independence [4]. In response, Virtual Wards (VWs) have emerged as an innovative model of home-based, multidisciplinary case management for individuals at high risk of hospitalization [8, 9]. The term ‘virtual’ refers to the delivery of hospital-level care in patients’ homes without a physical ward. Early models focused on chronic disease management, while more recent iterations emphasize acute frailty management through multidisciplinary teams and targeted rehabilitation [10, 11].

Community Virtual Wards (CVW), first developed in the UK and Ireland, aim to bridge primary and secondary care, reduce hospital admissions, and provide coordinated, person-centred care at home [12]. Research suggests that appropriate risk screening tools can optimize healthcare resources allocation for frail older adults [7].

Norway, with a population of 5.5 million, faces similar demographic challenges. The number of individuals aged 80 years or older is expected to more than double by 2050 [13]. Since the implementation of the Coordination Reform [14] there has been a deliberate shift of responsibility from specialist healthcare to primary care services [15], resulting in earlier hospital discharges and a growing demand for advanced follow-up in the community.

Despite national guidelines emphasizing holistic care pathways [16], primary care services often lack standardized frailty screening tools and structured assessments [4, 17, 18]. This represents a challenge in targeting the appropriate level of care for frail patients with complex needs.

To address these challenges, a municipality in northern Norway established a CVW in 2021. The CVW provides an alternative to institutional short-term care, emphasizing patient-centered, home-based rehabilitation for individuals transitioning from hospital to the community. Admission to the CVW is based on overall clinical condition rather than specific diagnoses.

To date, few studies have compared patient profiles, functional trajectories, and service needs between CVWs and traditional short-term care facilities such as Skilled Nursing Facilities (SNF), particularly in the Norwegian context [19, 20]. Most existing studies have focused on disease-specific populations or specialist-led hospital-at-home models [21–23] and to what degree hospital-at-home may provide similar outcomes to hospital admission [24].

This study aims to fill this gap by characterizing patient profiles in a municipality operated CVW and comparing them with those of patients admitted to a traditional SNF, assessing whether the CVW serves as a viable alternative to institutional care. In this study, we defined the viability of CVW as an alternative to institutional care by examining functional recovery (changes in ADL and CFS), service utilization (municipal service minutes), and clinical goal achievement.

## Methods

### Study design and setting

This longitudinal comparative cohort study was conducted to evaluate differences in patient profiles, functional recovery, and community service utilization for individuals transitioning from hospital care to either a Community Virtual Ward (CVW) or a Skilled Nursing Facility (SNF). The study took place in Bodø, a coastal city in northern Norway with approximately 55,000 inhabitants and a developed healthcare infrastructure, including digital home health initiatives. The Norwegian welfare model guarantees all inhabitants access to essential specialist and primary care, funded through municipal taxes. The system is designed to ensure that health services are accessible to everyone, regardless of where they live. The Ministry of Health and Care Services holds overall responsibility, while Regional Health Authorities oversee specialist care, and local municipalities are responsible for primary care and social services [15].

In Norway, acute medical treatment is provided in specialist hospitals. Once patients are medically stabilized but remain frail or functionally impaired, they may be discharged to municipal post-acute care. This care is provided either in a Skilled Nursing Facility (SNF; short-term municipal inpatient rehabilitation) or through a Community Virtual Ward (CVW; home-based follow-up serving as a substitute for institutional beds). In this study, “admission” refers to the first day in SNF or CVW following hospital discharge. Baseline measures of functional status (ADL and CFS) were collected at this time but reflect patients’ habitual level approximately three weeks prior to hospitalization. These data were retrieved retrospectively from patients’ medical records to avoid capturing temporary functional decline related to the acute illness itself.

### Description of the care models

The CVW, established in 2021, provides short-term, patient-centred care to individuals recovering at home, with a total capacity of 10 virtual beds. The CVW is staffed by a multidisciplinary team, including a dedicated physician (available weekdays 08:00–16:00), two nurses, a physiotherapist, and an occupational therapist. Care delivery includes home visits, video consultations and the use of sensor technology. Outside regular hours (evenings, weekends, holidays), traditional Home Care services and community emergency teams provide coverage.

The Skilled Nursing Facility (SNF) operates as a short-term inpatient unit offering advanced medical monitoring, rehabilitation services, and nursing care. With a total capacity of 74 beds across departments (10–11 beds per department, including palliative care, transitional care, urgent care, rehabilitation), it provides 24-hour care support by physicians, physiotherapists, occupational therapists, nurses and nursing assistants.

### Participants and recruitment

**Fig. 1:**
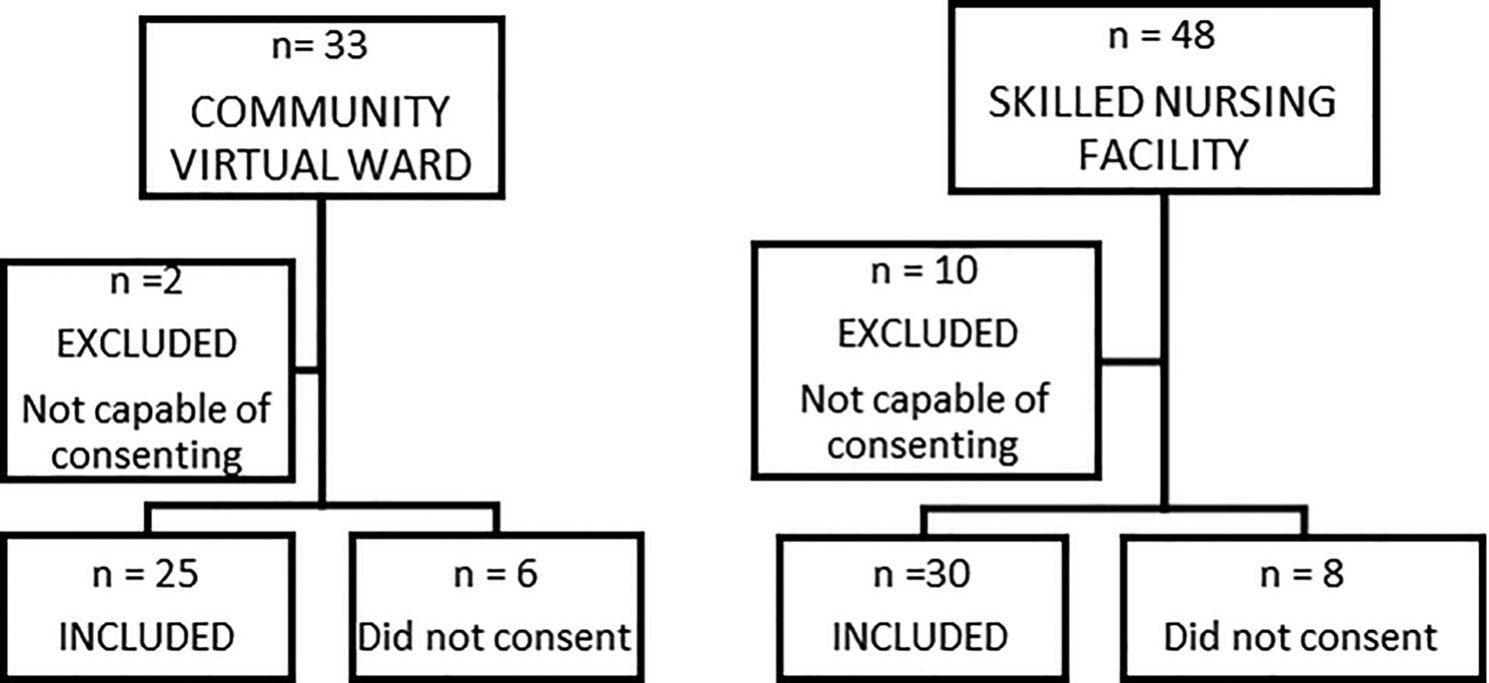
Recruitment flow chart. Participants identified *n* = 81

Adults aged 18 years or older who transitioned directly from hospital care to either the CVW or SNF between March and September 2023 were eligible. Exclusion criteria included: inability or unwillingness to provide informed consent, primary psychiatric diagnosis, and incomplete follow-up data at three months. A total of 81 patients were screened; 55 participants were included in the study, with 25 assigned to the CVW and 30 to the SNF (Fig. 1). Admission to the CVW was based on clinical judgment performed by the Municipal Allocation Office considering overall condition rather than specific diagnose or formal frailty thresholds. Allocation to CVW or SNF was therefore based on overall clinical stability, expected rehabilitation intensity, and the safety and suitability of the home environment; CVW was not a default destination.

### Data collection and measures

Data were collected at three time points: admission (reflecting habitual status three weeks prior to hospitalization), discharge, and 90 days post-discharge. Information was extracted from the municipality’s electronic patient record system and included: demographics (age, gender, housing situation), Clinical Frailty Scale (CSF) scores, Activities of Daily Living (ADL) scores, number and type of comorbidities and community service utilization (minutes per week allocated to practical assistance, home healthcare, rehabilitation). We used the Clinical Frailty Scale, version 2.0 [25] developed by Rockwood et al. [26] to summarize the overall level of fitness and frailty in patients based on mobility, function and cognition, and Activities of Daily Living scores developed by Katz et al. [27] to describe fundamental skills of everyday life including social and cognitive function, capacity of self-care and household activities. Both the CSF and ADL instruments are validated for use in elderly, community-dwelling populations. Patients were stratified into three age groups (< 65 years, 65–80 years, >80 years) to analyse differences in frailty trajectories, functional recovery, and service needs.

### Statistical analysis

Descriptive statistics summarized baseline characteristics. Continuous variables were analysed using t-tests or Mann-Whitney U tests, depending on normality, while categorical variables were compared using Chi-Square tests. Longitudinal changes CFS and ADL scores were assessed using paired t-tests, and group differences were evaluated through repeated measures ANOVA. Multivariate regression analyses were conducted to identify predictors of community service utilization at three months, adjusting for age, gender, comorbidities, group membership, and length of stay.

Collinearity among predictors was assessed; VIFs were elevated for ADL and CFS, while other predictors had lower values. Regression coefficients, confidence intervals, and p-values for all predictors are presented in Table 4.

Interaction terms (e.g., age × group membership) were explored but found non-significant. Missing data were handled through listwise deletion for regression analyses. No formal power calculation was performed, as this was an exploratory study with a limited target population. A two-sided p-value < 0.05 were considered statistically significant. Analyses were performed using IBM SPSS Statistics v.29 (IBM Corporation, Armonk, NY, USA).

### Ethical consideration

The study was approved by the Norwegian Agency for Shared Services in Education and Research (reference number 819933), the Regional Committee for Medical and Healthcare Research Ethics (reference number 533283), and the Municipality of Bodø’s local data protection officer. Written informed consent was obtained from all participants before enrollment.

## Results

A total of 55 patients were included in the study, with 25 admitted to the CVW and 30 to the SNF. The mean age of patients in the CVW group was 81.2 years (SD ± 7.2; range 64–97), compared to 83.6 years (SD ± 6.4; range 67–99) in the SNF group. The CVW group had a higher proportion of female participants (64%) compared to the SNF group (50%). Most patients in both groups lived in apartments, though this was more common among CVW patients (84% versus 63%). Suitable housing arrangements (defined as a home assessed by healthcare professionals to meet the individual’s functional needs—for instance, sufficient space for assistive devices) were slightly more frequent in the SNF group (70%) than in the CVW group (64%). A higher proportion of SNF patients (87%) were already recipients of primary healthcare services prior to admission compared to the CVW group (68%). Demographic characteristics are presented in Table 1.

**Table 1:** Study participants demographic characteristics

Characteristics	CVW (*n* = 25)	SNF (*n* = 30)
Age, (mean)	81.2 years (SD ± 7.2)	83.6 years (SD ± 6.4)
Male, n (%)	9 (36%)	15 (50%)
Female, n (%)	16 (64%)	15 (50%)
Living with a caregiver, n (%)	8 (32%)	13 (43%)
Living alone, n (%)	17 (68%)	17 (57%)
Primary healthcare recipients, n (%)	17 (68%)	26 (87%)
Mean, number of comorbidities	5.6	7.1
Mean, length of stay	15.5 days	29.6 days
Goal achievement, n (%)	20 (80%)	18 (60%)
Housing, n (%)		
• Apartment • Single-family house • Other	21 (84%) 3 (12%) 1 (4%)	19 (63%) 9 (30%) 2 (7%)
Suitable housing, n (%)		
• Yes • No • Unknown	16 (64%) 6 (24%) 3 (12%)	21 (70%) 1 (3%) 8 (27%)
Location		
• City center • Outskirts	13 (52%) 12 (48%)	17 (57%) 13 (43%)

n: Number of participants

CVW: Community Virtual Ward

SNF: Skilled Nursing Facility

Regarding comorbidities, patients in the SNF group had a greater burden, with a mean of 7.1 diagnoses (SD ± 2.6; range 3–12), compared to 5.6 (SD ± 2.64; range 1–12) in the CVW group. The most common primary reasons for admission across both groups were trauma/falls and infections (Table 2). Cardiovascular, metabolic, and musculoskeletal disorders were prevalent comorbidities in both cohorts (Table 3).

**Table 2:** Primary diagnosis

Primary diagnosis	CWV (*n* = 25)		SNF (*n* = 30)	
	n	%	n	%
Trauma/falls	10	40.0	8	26.7
Neurology	4	16.0	3	10.0
Infections	6	24.0	8	26.7
Gastrointestinal disorders	0	0	3	10.0
Oncology	1	4.0	2	6.7
Others	4	16.0	6	20.0

n: Number of participants

CVW: Community Virtual Ward

SNF: Skilled Nursing Facility

**Table 3:** Comorbidities

Comorbidities	CVW (*n* = 25)		SNF (*n* = 30)	
	n	%	n	%
Cardiovascular disease	37	26.2	54	25.2
Neurological disease	14	9.9	20	9.3
Metabolic disorders	20	14.2	29	13.6
Musculoskeletal disorders	20	14.2	23	10.7
Oncology	9	6.4	11	5.1
Respiratory disorders	7	5.0	8	3.7
Mental disorders	4	2.8	7	3.3
Others	30	21.3	62	29.0

n: Number of participants

CVW: Community Virtual Ward

SNF: Skilled Nursing Facility

At admission (reflecting habitual status three weeks prior to hospitalization), patients in the CVW group exhibited lower frailty scores, with a mean CFS of 4.2 (SD ± 1.62), compared to a mean of 5.3 (SD ± 1.29) in the SNF group. Functional independence as measured by ADL was also higher among CVW patients (mean ADL score 1.4, SD ± 1.08) than SNF patients (mean ADL score 2.5, SD ± 1.09).

During inpatient stay, both groups experienced increases in ADL scores, indicating greater functional dependence by discharge. In the CVW group, the mean ADL score increased to 2.2 (SD ± 0.85, *p* = 0.002), while in the SNF group it rose to 3.1 (SD ± 0.69, *p* < 0.001). Frailty scores also changed modestly during the admission period, with CFS increasing significantly in both the CVW (*p* = 0.003) and SNF groups (*p* < 0.001).

**Fig. 2:**
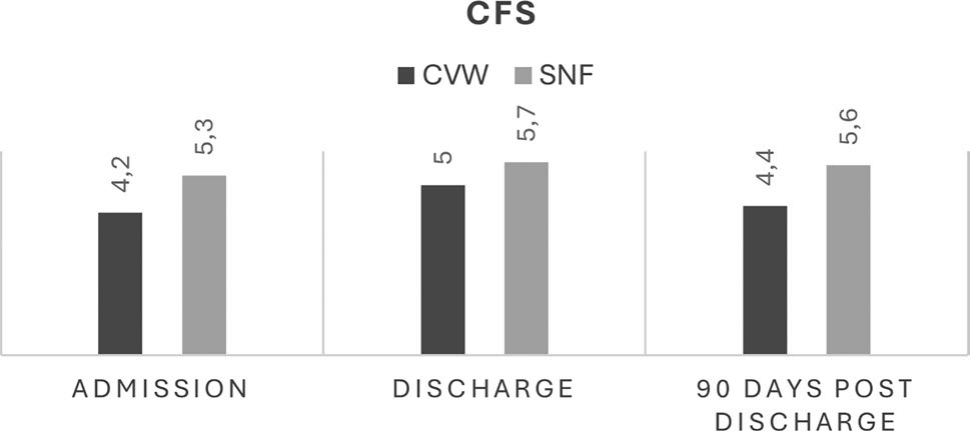
Average CFS-score at admission, discharge and 90 days post discharge. CFS: Clinical Frailty Scale score, CVW: Community Virtual Ward, SNF: Skilled Nursing Facility

**Fig. 3:**
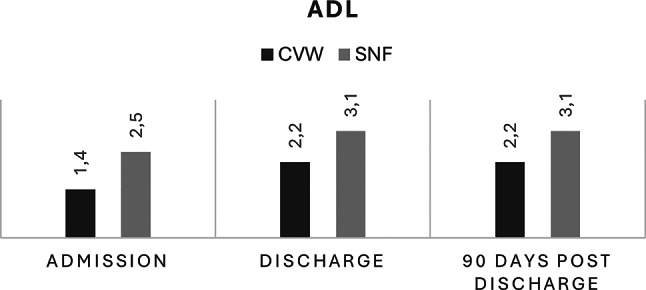
Average ADL-score at admission, discharge and 90 days post discharge. ADL: Activities of Daily Living score, CVW: Community Virtual Ward, SNF: Skilled Nursing Facility

At three months post-discharge, CVW patients demonstrated stabilization in functional independence, with mean ADL score of 2.19 (SD ± 0,87), and a small improvement in frailty, with a mean CFS score decreasing to 4.42 (SD ± 1.6). SNF patients exhibited a plateau in ADL scores (mean 3.06, SD ± 0.73) and persistent higher frailty levels (mean CFS 5.6, SD ± 1.3, Figs. 2 and 3).

Stratification by age groups (< 65, 65–80, and > 80 years) revealed that patients over 80 years had the highest baseline frailty (mean CFS 5.26, SD ± 1.29; *p* = 0.016; 95% CI 4.61–5.59) and required the most intensive community services, averaging 438 min of assistance per week (SD ± 374; 95% CI 312–565). In contrast, patients aged 65–80 years showed the greatest potential for frailty improvement, particularly among those admitted to the CVW. Although service use in this group averaged 199 min (SD ± 261; 95% CI 70–329), the difference in community service needs between groups was not statistically significant (*p* = 0.39).

**Fig. 4:**
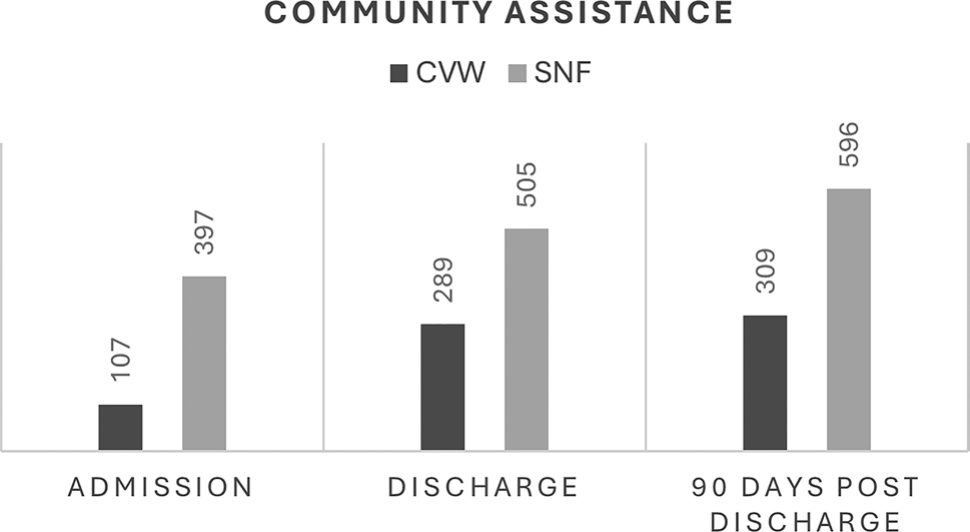
Average Time (minutes) of Community Services at admission, discharge, and 90 days post discharge. Community assistance: Weekly duration of community assistance per patient, measured in minutes, CVW: Community Virtual Ward, SNF: Skilled Nursing Facility

Community service utilization differed notably between groups. At admission, SNF patients received an averaging 397 min of community services per week (SD ± 366; 95% CI 318–476), compared to 107 min (SD ± 199; 95% CI 34–180) for CVW patients. By discharge, CVW patients’ service utilization increased to 289 min (SD ± 263; 95% CI 186–392), whereas SNF patients’ allocation rose more modestly to 505 min (SD ± 402; 95% CI 388–622). At three months, CVW patients required 309 min (SD ± 235; 95% CI 215–403), while SNF patients required 596 min (SD ± 457; 95% CI 460–732) of weekly services (Fig. 4).

In multivariable regression with 3-month community service minutes as the outcome, ADL at discharge and baseline frailty (CFS) emerged as the strongest predictors, although only ADL reached statistical significance. Specifically, ADL at discharge had a coefficient of 278.8 (95% CI 59.2–498.4, *p* = 0.014). Baseline frailty (CFS) was not statistically significant after adjustment (β = 89.9, 95% CI − 34.7 to 214.5, *p* = 0.152). Neither group membership (SNF vs. CVW; β = 8.2, 95% CI − 259.4 to 275.8, *p* = 0.949), length of stay (β = − 0.7, 95% CI − 8.3 to 6.9, *p* = 0.857), age (β = 6.1, 95% CI − 11.4 to 23.6, *p* = 0.486), nor sex (male vs. female; β = − 95.4, 95% CI − 321.6 to 130.8, *p* = 0.403) were significant predictors. Collinearity was observed between ADL and CFS, reflected in higher VIF values, while other predictors had lower values. Interaction terms, such as age and group membership (*p* = 0.364) and baseline CFS and group membership (*p* = 0.393), were tested but not found to be statistically significant (Table 4). These findings suggest that differences in downstream service use primarily reflect functional status at discharge rather than the care venue itself.

**Table 4:** Multivariable linear regression predicting community service minutes at 3 months

Predictor	Coefficient	95% CI	*P*-value
ADL at discharge	278.8	59.2–498.4	0.014
Baseline CFS	89.9	-34.7–214.5	0.152
Age (years)	6.1	-11.4–23.6	0.486
Length of stay (days)	-0.7	-8.3–6.9	0.857
Sex (male vs. female)	-95.4	-321.6–130.8	0.403
Group (CVW vs. SNF)	8.2	-259.4–275.8	0.949

ADL: Activities of Daily Living score

CFS: Clinical Frailty Scale score

CVW: Community Virtual Ward

SNF: Skilled Nursing Facility

Goal achievement rates, in terms of whether the patient met the treatment objectives defined by the Allocation Office, were higher in the CVW group (76.9%; 95% CI 55–91) compared to the SNF group (56.3%; 95% CI 38–72). This difference was not statistically significant (*p* = 0.3335). Mortality rates differed between groups during the three-month follow-up. Five patients in the SNF group and one patient in the CVW group died. Additionally, six SNF patients transitioned to long-term institutional care within three months post-discharge. These cases were excluded from the community service utilization analysis. Sensitivity analysis confirmed that their exclusion did not bias the results, as none had recorded data on community service use at three months.

## Discussion

This study compared the demographic profiles, functional recovery, and service needs of patients transitioning from hospital care to a Community Virtual Ward (CVW) versus a Skilled Nursing Facility (SNF) in a Norwegian municipality. The findings demonstrate that patients admitted to the CVW were generally characterized by mild frailty (CFS scores 4–5) compared to SNF patients, who exhibited moderate frailty levels (CFS scores 5–6). Mild frailty represents a transitional stage from independence to the need for regular assistance and was consistent with previous frailty categorizations described by Rockwood and Theou [25]. Functional independence, as measured by ADL, also showed better baseline levels among CVW patients compared to SNF patients. Group differences in frailty, ADL recovery, and community service use likely reflect case-mix from municipal triage: compared with SNF, CVW patients were younger and less frail at baseline and had shorter LOS (length of stay), consistent with lower subsequent service needs. Our adjusted analysis indicates that functional dependence at discharge (ADL) was the key independent driver of 3-month service allocation, underscoring that functional status, more than the immediate care venue, explains downstream resource use.

Patients in the CVW were slightly younger and had fewer comorbidities than those admitted to the SNF, aligning with previous research that identifies age and multimorbidity as significant drivers of frailty status [28, 29]. Goal achievement rates were higher among CVW patients, and their admissions were notably shorter. Moreover, CVW patients showed stabilization in functional dependence and slight improvement in frailty scores three months post-discharge, whereas SNF patients exhibited persistent higher frailty and functional dependency. However, while frailty scores (CFS) improved slightly in the CVW group, ADL scores worsened modestly, indicating increased functional support needs after discharge, consistent with findings from Lewis et al. [7] regarding recovery trajectories in frail populations.

The increase in allocated community services for both groups after discharge reflects the higher care needs of frail populations, consistent with the broader literature on community support post-hospitalization [7], and among individuals receiving Public Health Nursing services [30]. CVW patients experienced substantial increases in services during the inpatient period, likely reflecting the engagement of a multidisciplinary rehabilitation team. However, rehabilitation session frequency was not systematically recorded, limiting conclusions about the association between service intensity and functional outcomes, a factor emphasized in previous community-based intervention studies [31, 32].

Our findings correspond with international studies of CVW, particularly in Ireland, where frailty-focused interventions supported home-based recovery and reduced hospitalizations [7, 12]. However, the present study involved a mildly frail population, while Irish CVWs typically served individuals with more severe frailty (CFS 6–8). This difference suggests that CVW models can be adapted for varying degrees of frailty severity but also highlights the need for careful patient selection criteria [33] and markers of frailty in community dwellers [30]. Despite national guidelines encouraging holistic approaches to frailty in Norwegian primary care [16], routine use of standardized frailty tools remains inconsistent [18]. The absence of standardized assessments complicates optimal care placement decisions, as emphasized by Kim & Rockwood [17], Clegg et al. [3] and Dent et al. [4]. Internationally, systems such as the electronic Frailty Index [34] have demonstrated feasibility in integrating frailty assessments into primary care, yet adapting these tools to local contexts remains a challenge.

### Study limitations

This study has several limitations. First, the sample size was relatively small, and no formal power calculation was performed, limiting the detection of smaller differences between groups. Further, this was a single-centre study in a middle-sized municipality, limiting generalizability to broader settings, as similar limitations have been noted in previous frailty research [7, 35]. In addition, patients who died or entered long-term care during follow-up were excluded from certain analyses, which may have influenced community service utilization data, a challenge also highlighted in longitudinal studies of elderly populations [36]. Nonetheless, the results contribute valuable insights into the operationalization of frailty-focused transitional care pathways in primary healthcare.

### Implications for practice and research

This model aligns conceptually with the Hospital-at-Home movement in the United States, where home-based services substitute for institutional beds; our findings complement existing guidance and trials in that space. The findings suggest that Community Virtual Wards may serve as a viable intermediate care model for mildly frail individuals, offering an alternative to short-term institutional stays. Baseline frailty differed significantly between groups and appeared more influential than demographic factors in shaping recovery trajectories; however, in adjusted analyses only functional status at discharge (ADL) independently predicted service utilization at 90 days. These findings nonetheless support the integration of frailty screening into primary care practice [3, 37, 38], as baseline frailty remains an important marker for patient pathways. Future research should investigate the sustainability of outcomes in CVW patients, explore operational differences between CVWs modelled after SNFs versus hospital-at-home frameworks [12, 39], and further examine predictors of long-term dependency transitions.

Community-based screening and management have been introduced in some countries, e.g., Canada, France, Japan and China [4, 17, 40]. A 36-factor electronic frailty index using medical record data is being adapted to suit local conditions with the aim to build a frailty screening tool that will become a standard of practice in all Canadian primary care [34]. Since the benefit of routine frailty screening has not been consistently shown outside selected clinical settings [17], a unified approach is recommended to better measure and understand frailty at a wide range [23].

Hence standardizing frailty assessment in primary care as recommended by Clegg et al. [3], Ludlow et al. [37], Dejgaard and Rostoft [41], and the NICE guidelines [42] could enhance resource allocation and patient-centred care planning.

## Conclusion

This study identified notable differences between patients transferred from hospital care to a Community Virtual Ward (CVW) and those admitted to a Skilled Nursing Facility (SNF) within a Norwegian municipality. Patients admitted to the CVW were younger, had lower frailty scores, fewer comorbidities, and greater functional independence at baseline compared to SNF patients.

Across both groups, baseline frailty (CFS) differed significantly and was associated with recovery trajectories in descriptive analyses; however, in adjusted models only functional independence (ADL) at discharge independently predicted service utilization 90 days post-discharge. This is consistent with previous research underscoring the interplay between frailty and functional outcomes, while highlighting the central role of discharge ADL. In contrast, factors such as age, gender, group membership, and length of stay were not significant predictors of longer-term outcomes after adjustment for confounders.

These findings suggest that the CVW model may be a viable alternative to institutional short-term care for mildly frail individuals who benefit from rehabilitation-focused, home-based interventions, while the SNF remains indispensable for frailer patients requiring intensive and sustained support.

Although the findings support the growing role of intermediate care models such as the CVW, they also highlight a critical gap in Norwegian primary care: the absence of standardized frailty assessments. While routines for collecting data on frailty are recommended [42] implementation remains inconsistent. Strengthening the use of validated frailty-specific elements within primary care datasets could enhance patient selection, care planning, and resource allocation for aging populations with complex needs. Developing effective frailty detection and management strategies in primary care will be essential for ensuring that elderly individuals receive goal-directed, appropriate, and person-centred care across the continuum of services.

## Data Availability

The dataset generated and analyzed during the current study is not publicly available due to privacy regulations, but anonymized data is available from the corresponding author on reasonable request.

## Abbreviations

CVW: Community Virtual Ward
SNF: Skilled nursing facility
CFS: Clinical frailty scale
ADL: Activities of daily living
LOS: Length of stay
